# N7-methyladenosine-induced SLC7A7 serves as a prognostic biomarker in pan-cancer and promotes CRC progression in colorectal cancer

**DOI:** 10.1038/s41598-024-80885-2

**Published:** 2024-12-28

**Authors:** Fuqi Wang, Shiqian Zhang, Zhuang Chen, Xiaoming Gu, Ge Zhang, Hairong Zhang, Weitang Yuan

**Affiliations:** 1 Department of Colorectal Surgery, The First Affiliated Hospital of Zhengzhou University, No.1 Eastern Jianshe Road, Zhengzhou, 450052 Henan China; 2 Department of Cardiology, The First Affiliated Hospital of Zhengzhou University, Zhengzhou, China

**Keywords:** SLC7A7, Colorectal cancer, Migration, Invasion, m7g modification, Metastasis, Cancer, Cell biology, Genetics, Systems biology, Biomarkers, Gastroenterology, Health care

## Abstract

Solute transport family 7A member 7 (SLC7A7) mutations contribute to lysinuric protein intolerance (LPI), which is the mechanism of action that has been extensively studied. In colorectal cancer (CRC), SLC7A7 appears to play a role, but the features and mechanisms are not yet well understood. Survival was analyzed using the Kaplan–Meier analysis. Enrichment analysis was performed to characterize, immune infiltration, methylation, genetic instability, and crucial pathways of SLC7A7. Afterward, functional experiments were conducted in vitro to investigate how SLC7A7 affects tumor metastasis. Mechanistically, quantitative real-time PCR (qRT-PCR), western blot (WB), and methylated RNA immunoprecipitation (me-RIP) were carried out to confirm the methylation modification of SLC7A7 and related functions. High levels of expression of SLC7A7 are predictive of a worse prognosis for CRC patients. Enrichment analysis showed that SLC7A7 was significantly enriched during EMT and could be enriched in the Wnt/β-catenin signaling pathway, immune infiltration analysis of pan-cancer showed that SLC7A7 was significantly enriched in macrophages, and methylation analysis showed that SLC7A7 methylation modification affected the prognosis of specific cancers. SLC7A7 was indicated to promote the migration and invasion of CRC cells in in vitro functional experiments. Mechanistically, SLC7A7 was observed to potentially interact with the Wnt/β-catenin signaling pathway, possibly by influencing adenomatous polyposis coli (APC) expression. Furthermore, we identified that SLC7A7 undergoes N7-methylguanosine (m7G) modification, which may regulate SLC7A7 mRNA stability, with Quaking (QKI) potentially playing a role in this process by recognizing the m7G modification. Our results indicate that SLC7A7 may promote CRC metastasis through the SLC7A7/APC/Wnt/β-catenin signaling pathway. Moreover, m7G modification might be involved in regulating SLC7A7 mRNA stability, highlighting a novel layer of regulation.

## Introduction

CRC is a commonly occurring malignant tumor of the human digestive system, which maintains an increasing trend in morbidity and mortality^1,2^. Due to the low prevalence of colorectal early screening and the lack of obvious early symptoms, the majority of CRC patients are diagnosed at an advanced stage^3^. This group of patients still has a poor prognosis, particularly when they have advanced disease or distant metastatic spread^4^. Hence, it is crucial to elucidate the molecular mechanisms that lead to the occurrence and development of CRC and identify new biomarkers for early diagnosis and prediction, as well as new targets for the potent prevention and treatment of CRC recurrence and metastasis.

SLC7A7 is expressed predominantly in macrophages, enterocytes, and kidney cells^5,6^. The SLC7A7 gene encodes the y + LAT1 light chain of the y + L system, which binds to 4F2hc for the transmembrane transport of amino acids. It is thought that SLC7A7 mutations can lead to LPI, which exhibits symptoms such as hepatosplenomegaly, muscle hypotonia, and growth retardation^7,8^. There has been widespread research on the role of SLC7A7 in lysine urinary protein intolerance. Recent studies have revealed that SLC7A7 has other functions in addition to the arginine translocation of y + LAT-1. For example, SLC7A7 mutations lead directly to the production of the pro-inflammatory cytokines IL-1β and TNF-α, which contribute to lung disease^5,9^. In addition, SLC7A7 plays a crucial role in cancer initiation and progression. It has been shown that SLC7A7 is transcriptionally upregulated in hepatocellular carcinoma, causing a rise in arginine synthesis, and that high levels of cytosolic arginine are critical for liver tumor development^10^. It has been shown that high levels of SLC7A7 expression are associated with poorer survival and recurrence-free survival in lung cancer studies^11^. The expression of SLC7A7 in non-small cell lung cancer was also highly correlated with various immune marker groups^12^. In esophageal squamous cell carcinoma, SLC7A7 is highly expressed in tumor tissues and patients have a poor prognosis^13^. Nevertheless, there is still a lack of clarity about the potential function and mechanism of SLC7A7 in CRC.

The Wnt/β-catenin pathway comprises a family of proteins that play critical roles in embryonic development and adult tissue homeostasis. The deregulation of Wnt/β-catenin signaling often leads to various serious diseases, including cancer and non-cancer diseases. Wnt signaling is activated by binding to its receptor, which induces the binding of AXIN to phosphorylated lipoprotein receptor-related protein (LRP). The destruction complex(GSK-3, AXIN, CK1, APC) is broken, and then β-catenin stabilizes and binds to TCF in the nucleus to regulate the target gene^13^.

In this study, SLC7A7 was identified as a prognostic factor for CRC in multiple cohorts. Furthermore, we found that knockdown of SLC7A7 inhibited migration and invasion of CRC cells by activating the Wnt/β-catenin signaling pathway. SLC7A7 activated the Wnt/β-catenin signaling pathway, which may be owing to the APC. Moreover, we identified the presence of N7-methylguanosine (m7g) modification on SLC7A7 and that the degradation rate of SLC7A7 was regulated by the m7g recognition protein QKI. Our results indicate that SLC7A7 has the potential to promote tumor progression and serve as a potential therapeutic target for CRC.

## Materials and methods

### Data collection

CRC transcriptome and clinical data were sourced from the following databases: TCGA (https://portal.gdc.cancer.gov/), GTEx (https://www.gtexportal.org/) and GEO (http://www.ncbi.nlm.nih.gov/geo/).

### Survival analysis

The CRC patients in the TCGA and GEO cohorts were divided into the SLC7A7 high-expression group and the SLC7A7 low-expression group according to the optimal cutoff value of SLC7A7, and the Kaplan–Meier method and the log-rank test were used to analyze the overall survival (OS) and disease-free survival (DFS) of the CRC patients in the two groups.

### Enrichment and immune infiltration analysis

We used tools such as the DAVID online website (https://david.ncifcrf.gov/) to perform Gene Ontology (GO) enrichment analysis and Kyoto Encyclopedia of Genes and Genomes (KEGG) pathway analysis of SLC7A7^14^, and using Gene Set Enrichment Analysis (GSEA) to further explore the SLC7A7 in specific signaling pathway enrichment. Next, we analyzed immune infiltration using the CIBERSORT tool (CIBERSORTx, stanford.edu) to assess the correlation between SLC7A7 expression levels and immune cell infiltration, and analyzed normalized single-cell RNA sequencing data (nTPM) to assess the SLC7A7 in different cell types Expression level.

### SLC7A7 Expression is associated with methylation

Using DNA methylation data from the TCGA database, we analyzed the differences in methylation levels of SLC7A7 in different cancer types and normal tissues and analyzed the relationship between the methylation levels of SLC7A7 and OS and PFS in patients with different cancers, next, we evaluated the relationship between the methylation status of SLC7A7 and its mRNA expression levels.

### SLC7A7 genomic alterations

We analyzed the genomic variation of SLC7A7 in a variety of cancers using copy number variation (CNV) and single nucleotide variation (SNV) data from the TCGA database. We also used the COSMIC database to analyze the mutation types of SLC7A7 and to count the frequency of each type of mutation. Next, we analyzed the correlation between SLC7A7 CNV and mRNA expression levels and explored the effect of CNV levels on survival by analyzing the percentage of CNVs in unused cancers and the differences in OS between CNV groups.

### SLC7A7 genomic instability

CRC patients in the TCGA-CRC cohort were categorized into high SLC7A7 and SLC7A7 groups. As previously reported^15^, we analyzed the percent of mutation, gain, and loss in two groups, respectively.

### Cell culture

HCT116 cells and SW480 cells were obtained from the Shanghai Cell Bank of Chinese Academy of Sciences (Shanghai, China). HCT116 cells and SW480 cells were cultured at 37℃, CO_2_ 5% in an incubator, and cell culture flasks were supplemented with high sugar DMEM (HyClone, Logan, Australia) containing 10% fetal bovine serum (Biological Industries, Cromwell, CT, USA), and passaged in 48 h.

### RNA isolation, reverse transcription, and quantitative real-time PCR (qRT-PCR)

Total RNA was extracted using RNAiso Plus (Takara, Dalian, China), and after RNA extraction, NanoDrop One (Thermo Fisher Scientific, Waltham, USA) was used to determine the concentration of RNA and for quality control. Reverse transcription of RNA into cDNA and qRT-PCR were performed using QuantStudio 5 Real-Time PCR system (Applied Biosystems, Foster City, USA). qRT-PCR dye was SYBR and GAPDH as an internal reference.

The relevant primers are as follows.

The primer sequences were (5′-3′):

SLC7A7-forward CCTTTGGAGGATTCCTTGCTT.

SLC7A7-reverse GTTCCCCATTTGACATAGGCA.

QKI- forward AAGCCCACCCCAGATTACCT.

QKI- reverse ACTCTGCTAATTTCTTCGTCCAG.

APC-forward AAAATGTCCCTCCGTTCTTATGG.

APC-reverse CTGAAGTTGAGCGTAATACCAGT.

GAPDH- forward AACGGATTTGGTCGTATTGG.

GAPDH- reverse TTGATTTTGGAGGGATCTCG.

### Cell transfection

The siRNA and Lipofectamine 3000 (Thermo Fisher Scientific, Carlsbad, CA, USA) were mixed and incubated in DMEM and then transduced into HCT116 and SW480 cells, and the solution was changed after 6-8 h. siRNA-NC (siN0000001-1-5) and siRNAs-SLC7A7 (stB0008815A-1-5 and stB0008815B-1-5), si-QKI (siB170209021336-1-5) were purchased from Guangzhou RiboBio.

### Western blot analysis

Proteins from CRC cell lines were lysed with RIPA, total cellular proteins were obtained by centrifugation, and the proteins were processed at 100°C for 10 min after the addition of protein uploading buffer and detected by BCA Protein Detection Kit (Solarbio, Beijing, China). Equal amounts of proteins were separated by electrophoresis on SDS-PAGE gels and then transferred to PVDF membranes (Millipore, Massachusetts, USA). After blocking with a rapid blocking solution, the membrane was incubated with ZO-1(1:5000) (Proteintech, Wuhan, China), E-cadherin (1:20000) (Proteintech, Wuhan, China), β-catenin (1:5000) (Proteintech, Wuhan, China), Tubulin(1:3000) (Proteintech, Wuhan, China), QKI (1:1000) Proteintech, Wuhan, China), SLC7A7(1:3000) (Abcam, Cambridge, UK) and GAPDH primary antibodies (1:5000) (Proteintech, Wuhan, China) overnight at 4℃, washed with TBST, and then hybridized with the secondary antibodies for 1h at 37℃, washed again with TBST and exposed.

### Actinomycin D assay

SW480 cells were exposed to 200 ng/ml actinomycin D (Merck, Darmstadt, Germany) at 0h, 4h, 8h, 12h, 24h. Then the cells were harvested, and total RNA was extracted. The stability of SLC7A7 mRNA was analyzed using qRT-PCR.

### Wound healing and transwell assays

SW480 cells and HCT116 cells were transfected with siRNA for 48h for functional assays of invasion and migration. The transwell assay detected the invasive migration ability of cells, the treated cells and DMEM were added to the upper chamber, and after 24, and 36 h, the cells passing through the 8μm membrane were fixed, stained, and photographed. Transfected cells were inoculated into 6-well plates in the wound healing assay, and after the cells were attached to the wall after 24h and 48h, a cross-shaped scratch was formed on the 6-well plates with a 20μL pipette tip. Then, the scratches were photographed at 0, 24, and 48 h of wall attachment, respectively.

### me-RIP assay

The me-RIP assay was performed using the Magna me-RIP RNA-binding protein immunoprecipitation kit (Millipore, MA, USA) with anti-m7g antibody (MBL, Japan) and anti-immunoglobulin G (Millipore, MA, USA).

### Animal models

All animal experiments were performed in accordance and all mouse procedures were approved by the Institutional Animal Care and Use Committee of Zhengzhou University. Experiments complied with the ARRIVE guidelines, mice were anesthetized with isoflurane and euthanized by inhalation of excess carbon dioxide. BALB/c nude mice (4 weeks of age) were obtained from Vital River Laboratories (Beijing, China). After a one-week acclimation period, the mice were injected via the tail vein with stable HCT116 cells (2 × 10^6^ in 100 μL PBS) from each experimental group. On day 42, mice were sacrificed and lungs were collected for hematoxylin–eosin (HE) staining.

### Statistical analysis

Statistical analyses were performed using GraphPad Prism 9.0 (GraphPad, San Diego, CA, USA) and expressed as mean ± standard deviation. Discrete and categorical variables were compared between groups using Fisher’s exact test or chi-square test. Differences in continuous data were analyzed using the student’s t-test. Statistical significance was determined using the log-rank test, A two-sided p-value < 0.05 was considered statistically significant. Error bars represent 95% confidence intervals.

## Results

### SLC7A7 is associated with the expression and prognosis in pan-cancer

**Fig. 1:**
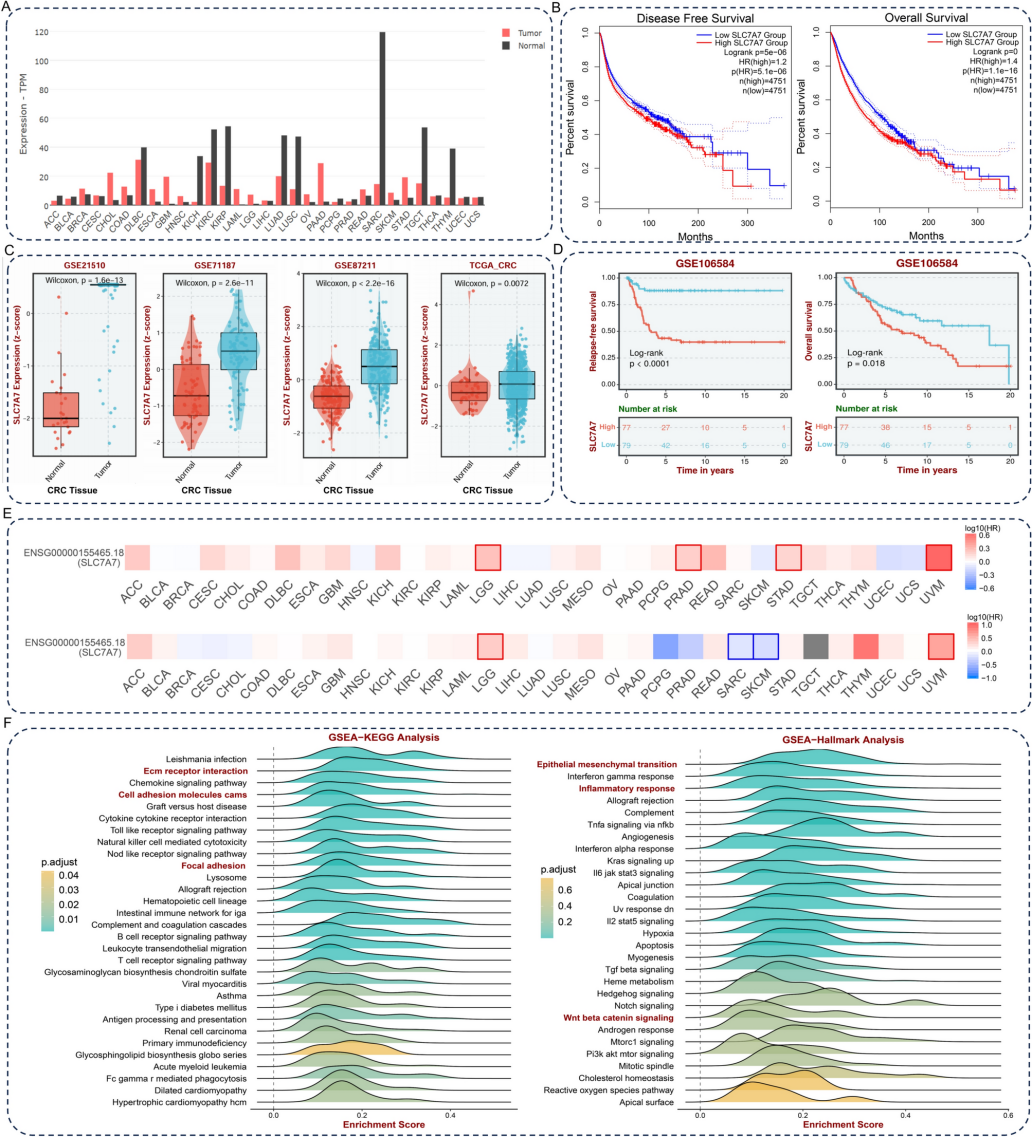
Diagnostic and prognostic values of SLC7A7 expression in pan-cancer. (**A**) The mRNA expression levels of SLC7A7 among 31 different human cancer tissues. (**B**) Kaplan–Meier survival analysis indicated that the OS and DFS of patients with high expression of SLC7A7 were all shorter than those of patients with low expression. (**C**) SLC7A7 expression in colon cancer tissues was analyzed by GEO and TCGA databases (GSE21510, GSE71187, GSE87211, and TCGA). (**D**) SLC7A7 expression was analyzed by GSE106584 concerning OS and RFS. (**E**) Survival maps show the prognosis of different tumors. (**F**). Enrichment of GSEA results based on SLC7A7 expression of the KEGG and Hallmark pathway.

We analyzed the mRNA expression levels of SLC7A7 among 31 different human cancer tissues. There was significant up-regulation of SLC7A7 mRNA expression in tumor tissues from Esophageal carcinoma (ESCA) Glioblastoma multiforme (GBM) Head and Neck squamous cell carcinoma (HNSC) Ovarian serous cystadenocarcinoma (OV) Pancreatic adenocarcinoma(PAAD), Skin Cutaneous Melanoma (SKCM), Stomach adenocarcinoma (STAD) and down-regulation in tumor tissues from Kidney Chromophobe(KICH), Kidney renal papillary cell carcinoma (KIRP), Lung adenocarcinoma (LUAD), Lung squamous cell carcinoma (LUSC), Testicular Germ Cell Tumors(TGCT) and Thymoma(THYM), as shown by data from the TCGA and GTEx databases (Fig. 1A). Meanwhile, we performed a survival analysis of these tumors, and Kaplan–Meier survival analysis indicated that the OS and DFS of patients with high expression of SLC7A7 were all shorter than that of patients with low expression (Fig. 1B). In CRC, the high mRNA expression level of SLC7A7 in CRC was validated by three independent GEO datasets and a TCGA dataset (Fig. 1C). In addition, the GSE106584 dataset validated that high expression of SLC7A7 was associated with poor OS and RFS (Fig. 1D).

The high expression level of SLC7A7 was associated with improved prognosis in Sarcoma (SARC) and SKCM but poor prognosis in BLGG and uveal Melanoma (UVM) as seen by survival maps. Meanwhile, high SLC7A7 expression levels were associated with short disease-free survival in LGG, STAD, UVM, and Prostate adenocarcinoma (PRAD) (Fig. 1E).

### Enrichment and immune infiltration analysis of SLC7A7 expression in CRC

**Fig. 2:**
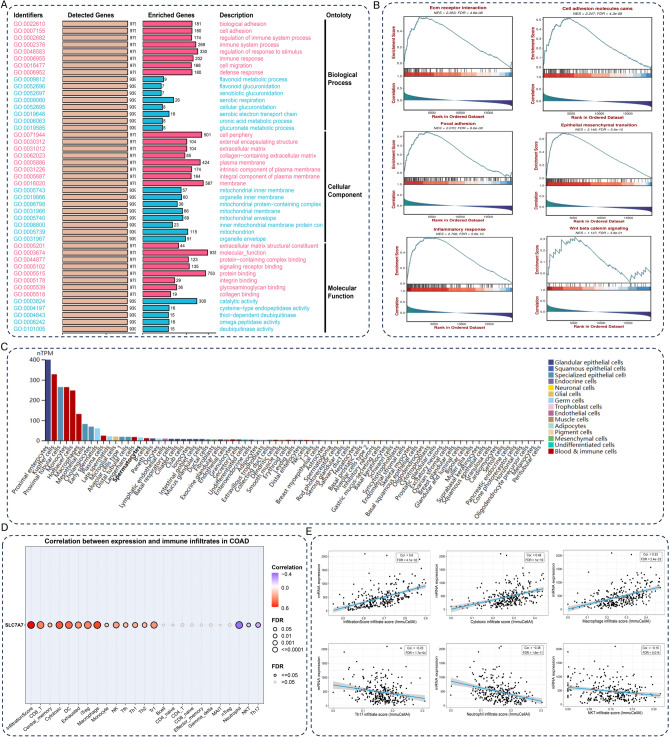
Enrichment and immune infiltration analysis of SLC7A7 expression in CRC. (**A**) Distribution is summarized into three broad categories: biological processes (BP), molecular functions (MF), and cellular components (CC). (**B**) Several important GSEA results are presented of SLC7A7 expression in GSEA pathways. (**C**) Summary of nTPM for all single-cell types, SLC7A7mrna Expression in Cells. (**D**) Analysis of SLC7A7 immune infiltration in colon cancer. (**E**) Listing of cells in the TCGA dataset positively and negatively correlated with SLC7A7mrna expression.

**Fig. 3:**
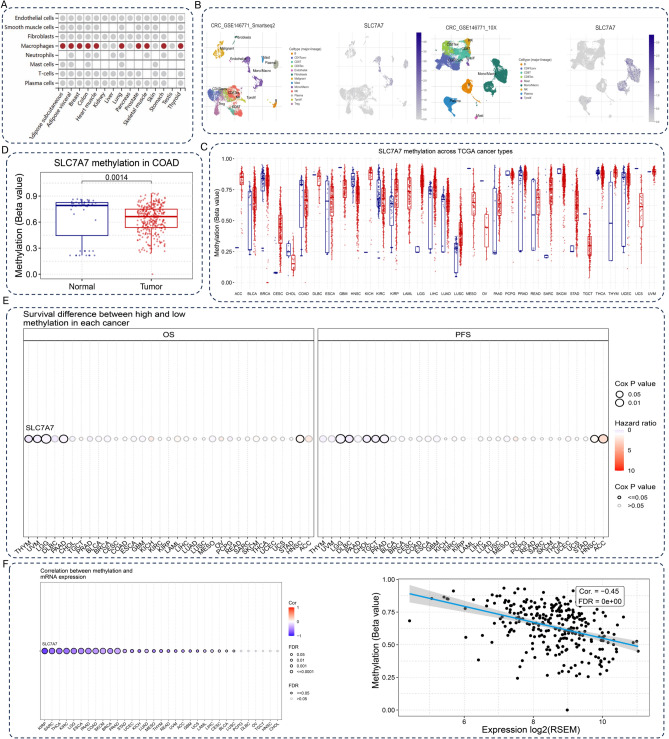
SLC7A7expression is associated with methylation in pan-cancer. (**A**) Enrichment analysis of SLC7A7 among eight immune cells in different tissues. (**B**) Single-cell sequencing results from colon cancer tissues were analyzed, and the vast majority of SLC7A7 was expressed in macrophages. (**C**) The expression of SLC7A7 methylation in different human cancer types. (**D**) The expression of SLC7A7 methylation in colorectal cancer. (**E**) Association of SLC7A7 methylation with prognosis in various cancers. (**F**) Methylation was negatively correlated with SLC7A7 expression in several tumors, including colon cancer.

To reveal the potential molecular mechanism of SLC7A7 in CRC, GO enrichment and KEGG pathway analysis were performed on SLC7A7. KEGG analysis showed that SLC7A7 was involved in Leishmania infection, extracellular matrix (ECM) receptor interaction, Chemokine signaling pathway, Cell adhesion molecules cams, Graft versus host disease, Cytokine receptor interaction, Focal adhesion, Nod-like receptor signaling pathway, Natural killer cell mediated cytotoxicity (Fig. 1F). GSEA − Hallmark Analysis shows that SLC7A7 was enriched in the Apical junction, Il6 jak stat3 signaling, Kras signaling up, Interferon alpha response, Angiogenesis, Tnfa signaling via nfkb, Complement, Allograft rejection, Inflammatory response, Interferon-gamma response, Epithelial-mesenchymal transition (EMT) (Fig. 1F). In Biological Process (BP), SLC7A7 is primarily responsible for the biological adhesion, cell adhesion, regulation of the immune system process, immune system process, regulation of response to stimulus, immune response, cell migration, and defense response. In the Cellular Component (CC), SLC7A7 was predominantly distributed in the cell periphery, external encapsulating structure, extracellular matrix, collagen − containing extracellular matrix, plasma membrane, an intrinsic component of the plasma membrane, integral component of plasma membrane, membrane. In Molecular Function (MF), which is mainly concerned with the ECM structural constituent, molecular function, protein − containing complex binding, signaling receptor binding, protein binding, integrin binding, glycosaminoglycan binding, and collagen binding (Fig. 2A). We also show several important GSEA pathways in Fig. 2B, these results indicate that EMT plays a critical role in the function of SLC7A7 in CRC. To study SLC7A7 mRNA expression, nTPM was pooled across all single-cell types. It was seen that in Proximal enterocytes, Kupffer cells, Proximal tubular cells, Monocytes, Hofbauer cells, and Macrophages, an elevated expression of SLC7A7 mRNA was observed (Fig. 2C). The correlation between SLC7A7 expression and the level of immune cell infiltration was assessed with an immune infiltration assay, tumor tissues with higher SLC7A7 expression levels were accompanied by higher Infiltration Score, CD8_T cells, Central memory cells, Cytotoxic cells, DC cells, Exhausted cells, iTreg cells, Macrophage cells, Monocyte cells, NK cells, Tfh cells, Th1 cells, Th2 cells, Tr1 cells. Tumor tissues with higher SLC7A7 expression levels were accompanied by lower Neutrophil cells, NKT cells, and Th17 cells (Fig. 2D). The expression level of SLC7A7 has different correlations in the immune pathways of various cells, such as a positive correlation between immune infiltration and cytotoxicity scores, and a negative correlation between neutrophils and Th17 infiltration scores (Fig. 2E). Further to investigate the role of SLC7A7 in immunity, the expression of eight immune cells in tissue cells was analyzed, SLC7A7 is highly expressed in macrophages in Adipose subcutaneous, Adipose, Visceral, Breast, Colon, Heart muscle, Lung, Prostate, Skeletal muscle, Stomach, Thyroid (Fig. 3A). Single-cell sequencing results of colon cancer tissues were analyzed, and the vast majority of SLC7A7 was expressed in macrophages (Fig. 3B). Taken together, these results indicate that the expression level of SLC7A7 in cancer is closely related to the infiltration of multiple immune cells.

### SLC7A7 expression is associated with methylation in pan-cancer

RNA methylation is implicated in human development, immunity, tumorigenesis and metastasis, stem cell renewal, adipose differentiation, and other processes. We analyzed the expression of SLC7A7 methylation in different human cancer types, tumor tissues, and normal tissues in the TCGA dataset (Fig. 3C) in CRC, SLC7A7 methylation is hyper-expressed within the tumor tissue (Fig. 3D). We also analyzed the relationship between methylation and OS, and the high expression level of SLC7A7 methylation affected OS in THYM, UVM, LGG, PAAD, and HNSC; PFS in LGG, DLBC, CHOL, TGCT, PRAD, HNSC, and ACC (Fig. 3E). Methylation was negatively correlated with SLC7A7 expression in several tumors, including colon cancer (Fig. 3F).

### SLC7A7 genomic alterations and genomic instability

**Fig. 4:**
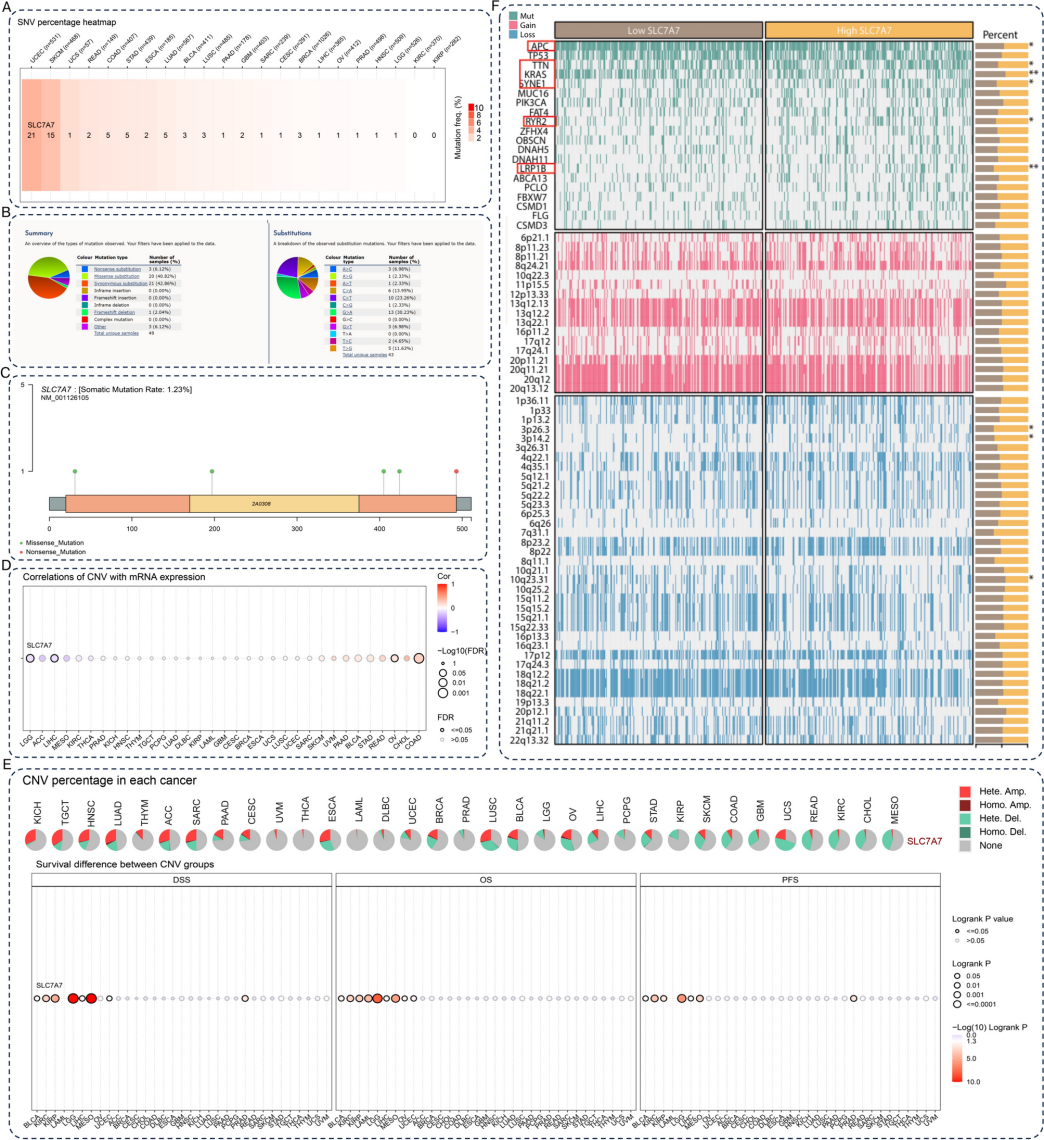
SLC7A7 genomic alterations and genomic instability. (**A**) SLC7A7 SNV were analyzed for pan-cancer. (**B**) Evaluated the COSMIC database for mutation types in SLC7A7. (**C**) In colon cancer, the SLC7A7 Somatic Mutation Rate: is 1.23%. (**D**) Correlations of CNV with mRNA expression. (**E**) CNV percentage in each cancer and Survival difference between CNV groups. (F) Variation in gene mutation, Gain, and Loss of high and low SLC7A7 expression in colon cancer.

To assess potential genome-level alterations of SLC7A7 in specific cancers, the SLC7A7 CNVs and SNVs were analyzed for pan-cancer. SLC7A7 SNV incidence is evident in UCEC, SKCM, COAD, and STAD (Fig. 4A). Analysis of SLC7A7 mutation types by COSMIC database, among the 503 samples with SLC7A7 mutation, Nonsense substitution of 14 (2.78%), Missense substitution of 153 (30.42%), Synonymous substitution of 94 (18.69%), and 1 Frameshift insertion (0.20%) occurred (Fig. 4B). In colon cancer, the SLC7A7 Somatic Mutation Rate: is 1.23% (Fig. 4C). Correlations of CNV with mRNA expression show that CNV was positively correlated with SLC7A7 mRNA expression in OV, and COAD and negatively correlated with SLC7A7 mRNA expression in LGG and LIHC (Fig. 4D). At the same time, we analyzed the CNV percentage in each cancer and the OS difference between CNV groups, and the results suggested that CNV levels influenced survival (Fig. 4E). We analyzed the changes in SLC7A7 and gene mutations, acquisitions, and deletions in colon cancer, the difference in APC, TTN, KRAS, SYNE1, RYR2, and LRP1 mutations was statistically significant (Fig. 4F). This indicates that the destabilizing genes may play a key role in cancer development.

### SLC7A7 promotes CRC invasion and migration in vitro and in vivo

**Fig. 5:**
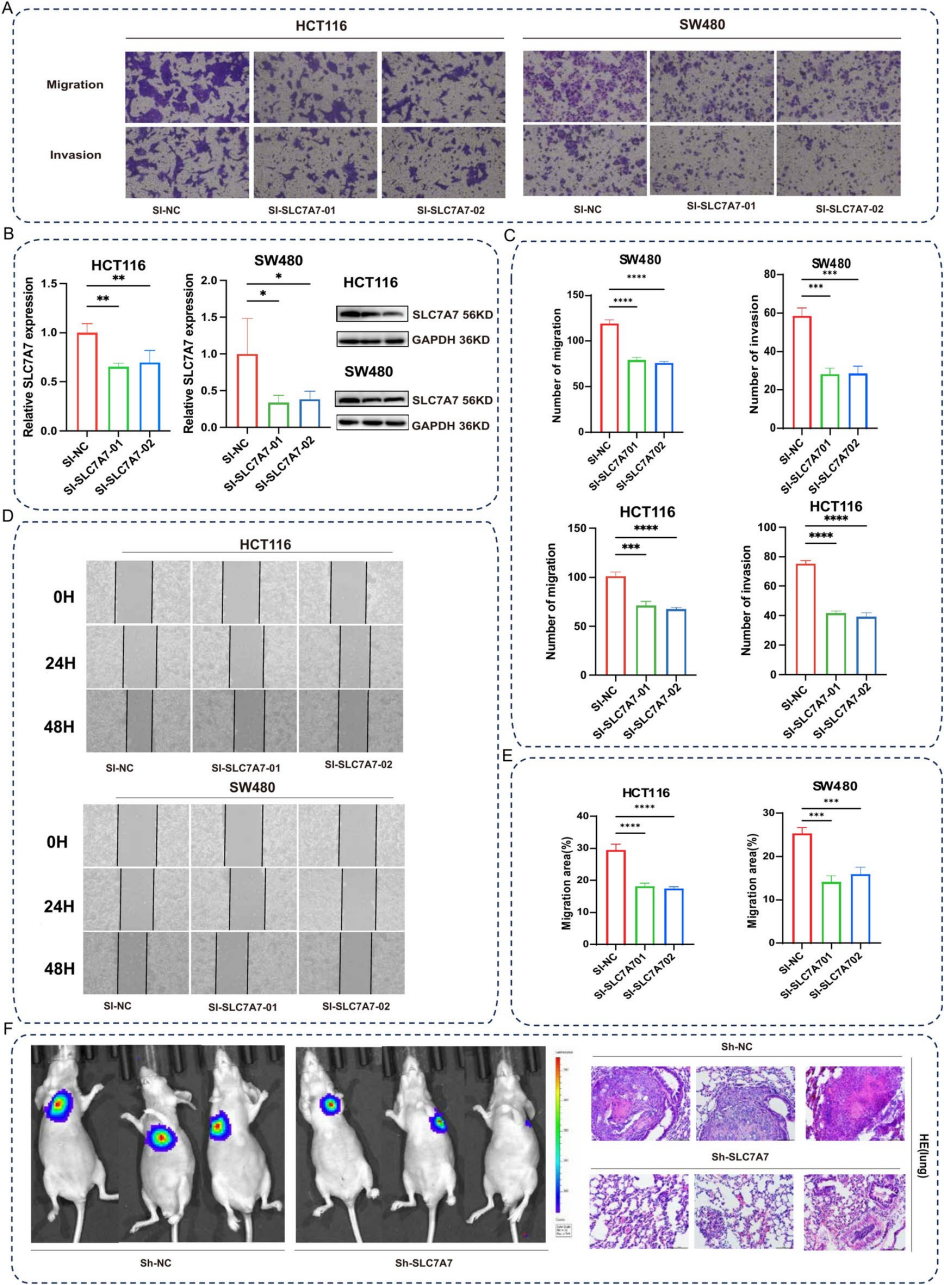
(**A**)SLC7A7 promotes CRC invasion and migration in vitro and in vivo. Transwell shows that after the knockdown of SLC7A7, both invasion and migration are reduced compared to NC. (**B**) The efficiency of SLC7A7 knockdown by qRT-PCR and WB in HCT116 and SW480 cell lines. (**C**) The statistic graph of invasion and migration in HCT116, SW480 cell lines transwell statistic graph. (**D**) Wound healing assay of HCT116 and SW480 cell lines at 0 h, 24 h, and 48 h, compared to the NC group, siSLC7A7 resulted in diminished cell migration. (**E**) The migration statistic graph of wound healing assay in HCT116 and SW480 cell lines. (F) Bioluminescent images of lungs for each experimental group. HE staining of lung sections displayed metastatic nodules of the lungs (magnification, × 100, scale bar,100 μm).

To explore the biological function of SLC7A7 in CRC cells, the siRNA against SLC7A7 was constructed. Transwell assays indicated that knockdown of SLC7A7 reduced the number of invasive and migratory cells (Fig. 5A,C). As shown in Fig. 5B& Supplemental fig1 the siRNA of SLC7A7 effectively reduced the expression of SLC7A7 in HCT116 and SW480 cells. We investigated the effects of SLC7A7 on the migratory and invasive abilities of CRC cells. Similarly, wound healing assay results revealed that there was a diminution in the migratory area after 48h of culture in the si-SLC7A7 cells compared to si-NC cells (Fig. 5D,E). To determine the effects of SLC7A7 on tumor metastasis in vivo, stably transfected HCT116 cells infected with Sh-NC or Sh-SLC7A7 were constructed and tail vein injected into 4-week-old female nude mice. Moreover, HE staining showed that downregulation of SLC7A7 significantly reduced lung metastases (Fig. 5F). Our results indicate that SLC7A7 promotes the migration and invasion of CRC.

### SLC7A7 activation of the Wnt/β-catenin signaling pathway promotes colon cancer progression through the EMT process and m7g modification promotes SLC7A7 mRNA stabilization.

**Fig. 6:**
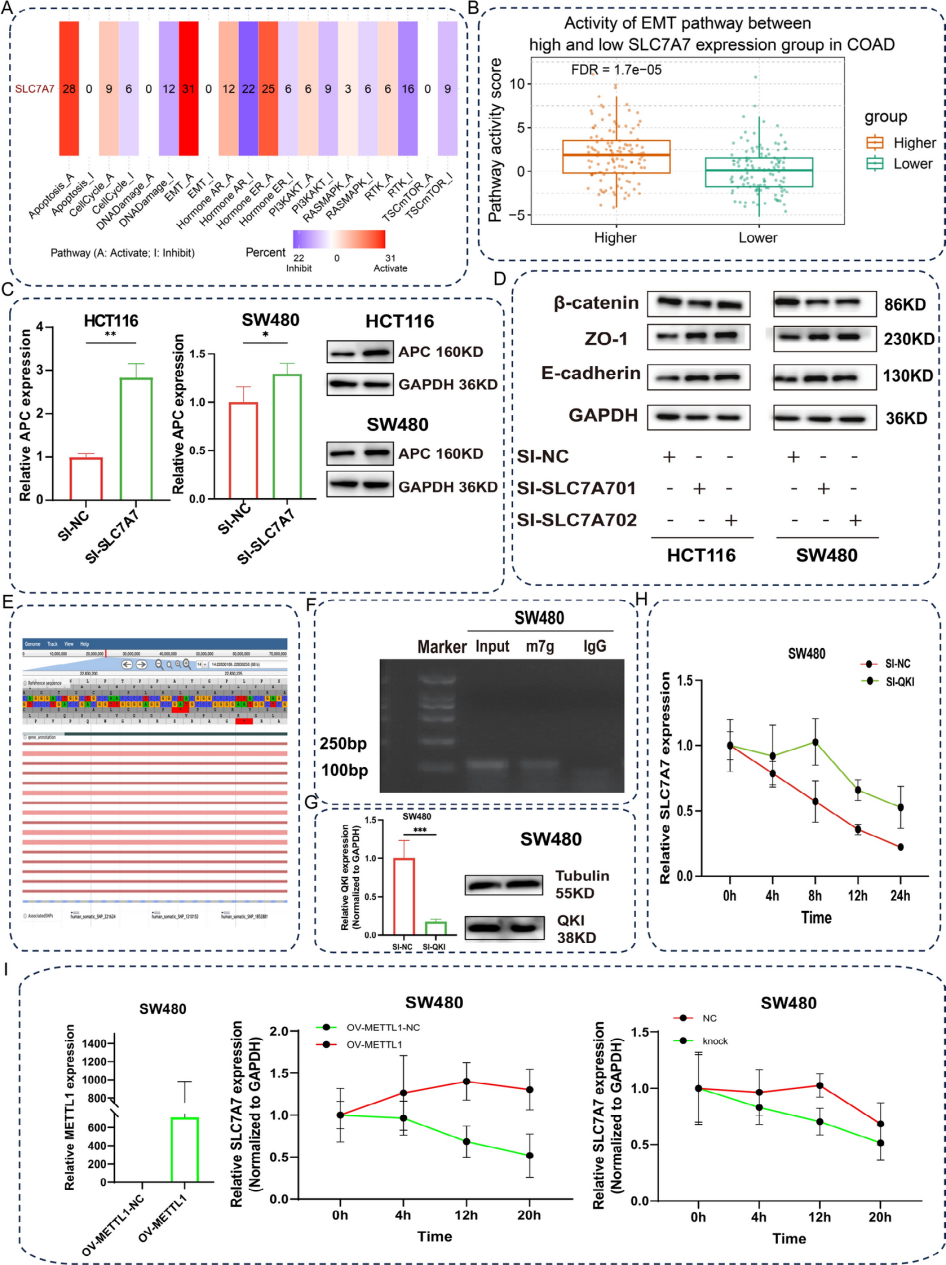
SLC7A7 activation of Wnt/β-catenin signaling pathway promotes colon cancer progression through the EMT process and m7g modification promotes SLC7A7 mRNA stabilization. (**A**) Enrichment analysis results showed that SLC7A7 significantly enriched in EMT Apoptosis Hormone ER et al. (**B**) Activity of EMT pathway between high(n = 243) and low(n = 243) SLC7A7 expression groups in COAD. (**C**) The APC expression of si-SLC7A7 was analyzed by qRT-PCR and WB in HCT116 and SW480 cells. (**D**) The expression of E-cadherin, ZO-1 β-catenin in HCT116 and SW480 cells transfected with SLC7A7 siRNA was detected by Western blot. (**E**) The m7g-hub website predicts the SLC7A7 mRNA m7g modification site. (**F**) The m7g antibody specifically enriches SLC7A7 mRNA. (**G**) The efficiency of QKI siRNA was detected using qRT-PCR and WB in SW480 cells. (**H**) Analysis of SLC7A7 mRNA levels in down-regulated QKI SW480 cells incubated with actinomycin D for 0 h, 4 h, 8 h, 12 h, and 24 h by qRT-PCR. (**I**)The efficiency of OV METTL1 was detected using qRT-PCR, analysis of SLC7A7 mRNA levels in overexpression METTL1 SW480 cells incubated with actinomycin D for 0 h, 4 h, 12 h, and 20 h by qRT-PCR.analysis of SLC7A7 mRNA levels in OV-METTL1-mutant SW480 cells incubated with actinomycin D for 0 h, 4 h, 12 h, and 20 h by qRT-PCR.

To understand the molecular mechanisms by which SLC7A7 regulates CRC progression, we’ve examined the relationship between SLC7A7 expression and gene alteration. Among the CRC gene mutations, most of the genes showed non-significant differences in expression levels between the low and high SLC7A7 groups. However, higher incidence of APC, TP53, KRAS, SYNE1, RYR2, and LRP1B mutations among CRC patients who express higher levels of SLC7A7. As mentioned above, SLC7A7 can be oncogenic in CRC through these gene alterations. Enrichment analysis results showed that SLC7A7 significantly enriched in EMT (Fig. 6A, B), and the WB was performed to detect changes in E-cadherin and ZO-1 in response to SLC7A7 downregulation (Fig. 6D & Supplemental Fig. 3). Therefore, we measured APC mRNA in SLC7A7 knockdown HCT116 and SW480 cells. The results indicated that the expression of si-SLC7A7 was significantly higher in HCT116 and SW480 cell lines compared to the NC group, and the WB results similarly suggest that the expression of si-SLC7A7 was significantly high (Fig. 6C & Supplemental Fig. 2). In the si-SLC7A7 group, E-cadherin and ZO-1 were noticed to be increased compared to the si-NC group. Similarly, SLC7A7 can be enriched in the Wnt/β-catenin signaling pathway, we detected changes in the β-catenin protein by western blot experiment, and β-catenin decreased with the knockdown of SLC7A7. N7-methylguanosine modification participates in multiple mRNA disorders that impact tumor regression. Check the SLC7A7 for the existence of the m7g modifier via the m7g-hub website (Fig. 6E). According to the results of the me-RIP, the anti-m7g antibody concretely enriched SLC7A7 mRNA in SW480 cells (Fig. 6F & Supplemental Fig. 4). In this case, it indicated the presence of m7g modification on SLC7A7 mRNA. Recruitment of the m7g reader protein to regulate the fate of mRNAs modified with m7g. To determine that QKI regulates the metabolism of SLC7A7 mRNA, we knocked down the expression of QKI in SW480 cells through transfection of QKI siRNA (Fig. 6G & Supplemental Fig. 5). Subsequently, actinomycin D assay was performed. The results indicated that the knockdown of QKI markedly slowed down the degradation of SLC7A7 mRNA (Fig. 6H). These results indicate that the degradation of SLC7A7 mRNA may be mediated by the m7g reading protein QKI. To further understand whether the m7g modification is involved in the degradation of SLC7A7 mRNA, we overexpressed METTL1, Subsequently, an actinomycin D assay was performed. The results indicated that overexpressed METTL1 markedly slowed down the degradation of SLC7A7 mRNA. At the same time, we constructed the OV-METTL1-mutant plasmid (METTL1 mutation site 1: aa107-109, EIR mutated to AAA), and actinomycin D assay was performed, the results indicated that the OV-METTL1-mutant markedly increased the degradation of SLC7A7 mRNA (Fig. 6I). These results indicate that the m7g modification was involved in SLC7A7 mRNA degradation.

## Discussion

The role of SLC7A7 in arginine metabolism has been extensively studied. With the extensive research on the molecular mechanisms of tumorigenesis and development, the role of SLC7A7 in tumorigenesis and development has gradually increased.

In CRC patients, upregulation of SLC7A7 in tumor tissues was significantly related to lower survival. It is similar to esophageal squamous cell carcinoma (ESCC), SLC7A7 was expressed higher than in normal tissues^13^. SLC7A7 was up-regulated in LGG and was associated with unfavorable prognosis in LGG patients^16^. However, in a study of NSCLC, SLC7A7 was indicated to be involved in the infiltration of neutrophils, tumor-associated macrophages (TAMs), and DCs in a variety of cancers, as well as in the regulation of T-cell exhaustion and Tregs in NSCLC^12^. Similarly, in our study, SLC7A7 was up-regulated in CRC and was also associated with an unfavorable prognosis.

Enrichment analysis indicated that SLC7A7 was positively associated with ECM receptor interactions, focal adhesion, EMT, and cell adhesion molecule cam. These results suggest that SLC7A7 may regulate CRC progression through the EMT process. E-cadherin and ZO-1 were quintessential indicators of EMT. Knocking down SLC7A7 expression in HCT116 and SW480 cells. We can observe that E-cadherin and ZO-1 were upregulated in CRC cells. Our study found that SLC7A7 was associated with the expression of multiple EMT marker genes in CRC, suggesting that it may play an important role in regulating the EMT process^17,18^. In addition, bioinformatics analysis revealed that SLC7A7 may promote EMT by activating the Wnt/β-catenin signaling pathway, a pathway known to be closely associated with tumor cell migration and invasion. Although the results of functional experiments supported the promotional effect of SLC7A7 on CRC cell migration and invasion, further experiments are needed in the future to validate the specific mechanism by which SLC7A7 regulates EMT through the Wnt/β-catenin signaling pathway. These findings suggest that SLC7A7 could be a potential therapeutic target, and the EMT process could be curbed by inhibiting its function, thus preventing the progression and metastasis of CRC.

In our study, we found that the methylation level of SLC7A7 was significantly elevated in CRC tumor tissues, and this increased methylation was negatively correlated with the expression level of the SLC7A7 gene, implying that methylation may play an important role in the pathogenesis of CRC by silencing gene expression. This negative correlation between methylation and gene expression suggests that SLC7A7 methylation in CRC may influence tumor progression by suppressing its expression^19^. This is consistent with the known role of DNA methylation as a mechanism of gene silencing, especially in the inactivation of oncogenes and activation of oncogenes, where methylation modifications usually play a key role^20^. Thus, methylation of SLC7A7 may not be merely a concomitant phenomenon but is involved in the development and progression of CRC. Further comparative analyses revealed that although methylation of SLC7A7 occurs in multiple cancer types, its elevation in CRC is specific. This specific elevation may be associated with the unique molecular background of CRC, such as specific oncogenic signaling pathways, unique immune responses in the tumor microenvironment, or other specific epigenetic regulatory mechanisms^21^. This suggests that the role of SLC7A7 methylation in CRC may be different from that in other cancers and may provide new insights into the pathogenic mechanisms specific to CRC.

The alteration of the genome, such as mutation, loss, and gain of genes, plays an important role in tumors. Among CRC, one oncogene (KRAS) and three tumor suppressor genes (APC, SMAD4, and TP53) are the main genetic changes^22^. In our study, we compared the genetic alterations in patients with differently expressed SLC7A7. Together, we found that the differences in APC, and KRAS mutations were statistically significant between the two groups. Mutations in the APC gene are not only responsible for familial adenomatous polyposis (FAP) but also play a rate-limiting role in the majority of sporadic CRC^23–26^. Loss of APC gene function seems to trigger the cascade of events that eventually lead to malignant transformation in the large bowel. We showed that SLC7A7 can affect the process of EMT, which is regulated by various signaling pathways, such as TGF-β, Wnt/β-catenin, Hedgehog, and Notch signaling pathways, and we also found that knocking down SLC7A7 expression increased APC mRNA expression, and combined with the biosignature analysis we believe that SLC7A7 can affect the EMT process by affecting the APC mRNA expression. We know that APC also represents a very important component of the Wnt signal transduction pathway ^21–23^. Under normal conditions, negative regulators of the Wnt/β-catenin signaling pathway, such as APC, glycogen synthase kinase 3 protein (GSK3), and axin, bind to β-catenin to form a complex that undergoes phosphorylation and further degradation^27^. Meanwhile, enrichment analysis also confirmed that SLC7A7 could be enriched in the Wnt/β-catenin signal pathway. Thus, we detected changes in β-catenin protein, which in combination with APC expression predicted that SLC7A7 could affect the Wnt/β-catenin signaling pathway through changes in APC mRNA expression.

The Wnt pathway plays an important role in EMT^28^. In the classical Wnt pathway, extracellular Wnt proteins bind to the Frizzled receptor on the cell membrane, activate the degradation protein DVL1, inhibit the phosphorylation of GSK3β, and cause the accumulation of β-catenin in the cytoplasm, which then transported to the nucleus,

where it binds to the promoter region of the target genes, such as Snail1 and activates their transcription, together with TCF, LEF and Snail1, etc., in the promoter region and activate their transcription^29^. These transcription factors inhibit the expression of epithelial cell-related genes while promoting the expression of mesenchymal cell-related genes, thus promoting the transition of cells to the mesenchymal state.

The m7G-cap, catalyzed by RNA guanine-7 methyltransferase during transcription initiation, protects mRNAs from exonucleolytic decay and modulates pre-mRNA splicing and mRNA translation^30,31^. Meanwhile, tRNAs7 and rRNAs8 are also heavily m7G-modified^32,33^. We found the presence of m7g modification in SLC7A7 mRNA by me-RIP. According to a recent report, researchers screened and identified QKI, the first recognition protein for internal m7G-modified mRNAs, and reported that internal m7G mRNAs are selectively recognized by QKI proteins and that QKI dynamically modulates m7G-modified mRNAs in cellular stress granules under stress conditions^34^, so we performed actinomycin D experiments. The results indicated that the degradation of SLC7A7 mRNA was slowed down following the knockdown of QKI. The regulators of m7G modifications are m7G methyltransferases, whose primary function is to add m7G modifications to target RNAs, thereby affecting RNA production, structure, and maturation, and ultimately mediating a variety of key biological processes. The best characterized m7G regulators are METTL1 and WDR4, which form a complex to catalyze the modification of various types of m7G RNAs.so we performed actinomycin D experiments. The results indicated that overexpressed METTL1 markedly slowed down the degradation of SLC7A7 mRNA. Mutated METTL1 action site markedly increased the degradation of SLC7A7 mRNA It is suggested that the m7g modification is involved in the biological process of CRC ^34^.

Several limitations should be noted, although we focused on analyzing the role of QKI in RNA methylation, the current evidence mainly relies on qRT-PCR and WB, etc., which needs to be further validated by methods such as single-cell sequencing and spatial transcriptome. In addition, the exploration of other RNA methylation-related proteins in the study was more limited, and future work could be extended to more related proteins to fully understand their functions in CRC.

## Conclusion

In conclusion, we explored the diagnostic prognostic value of SLC7A7 in pan-cancer utilizing multiple bioinformatics analyses. We also explored the role of SLC7A7 methylation in tumors, and we verified that m7g modification is involved in regulating SLC7A7 mRNA degradation in CRC. Subsequently, we demonstrated through functional and molecular biology experiments that SLC7A7 promoted migration and invasion of CRC cells may through the SLC7A7/APC/Wnt/β-catenin signaling pathway. These findings provide new ideas for SLC7A7 as a potential therapeutic target for CRC.

## Supplementary Information

Supplementary Figures.

## Data Availability

The data sets analyzed in this study are available in the GEO database, TCGA database, and GTEx database. All of the multiple microarrays are derived from these databases.
