# Supplementary material for: N7-methyladenosine-induced SLC7A7 serves as a prognostic biomarker in pan-cancer and promotes CRC progression in colorectal cancer
Source: Sci Rep. 2024 Dec 28;14:30755. doi: 10.1038/s41598-024-80885-2 (PMC11680768; doi:10.1038/s41598-024-80885-2)
Supplement: Supplementary file 1 — Supplementary Figures. [file 41598_2024_80885_MOESM1_ESM.docx]

**N7-methyladenosine-induced SLC7A7 serves as a prognostic biomarker in pan-cancer and promotes CRC progression in colorectal cancer**

**Supplementary Figures**


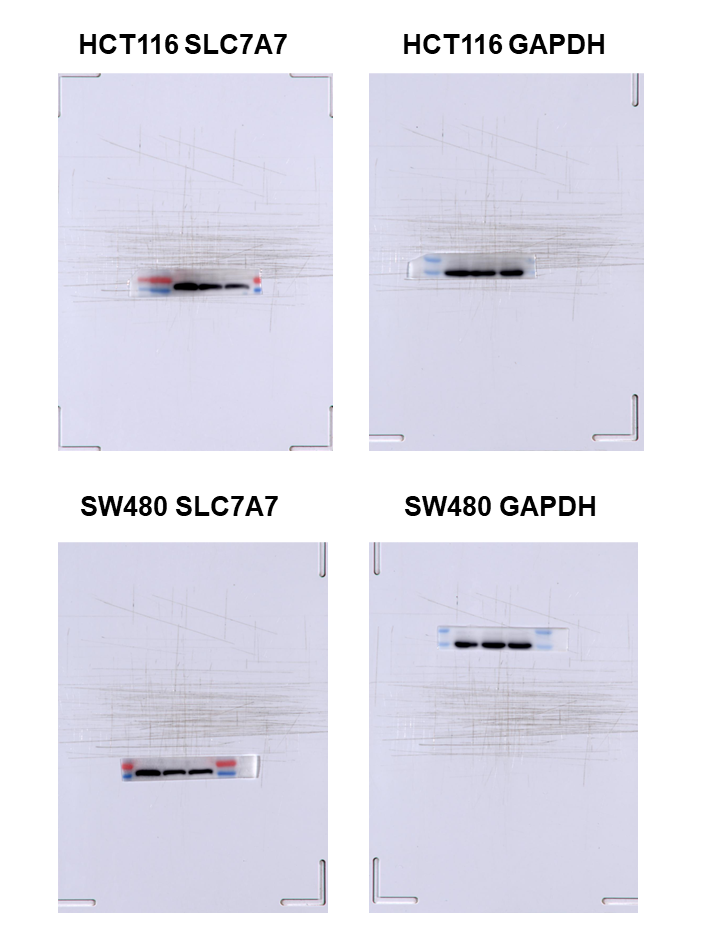


**Supplemental Figure 1: The efficiency of SLC7A7 knockdown by WB**


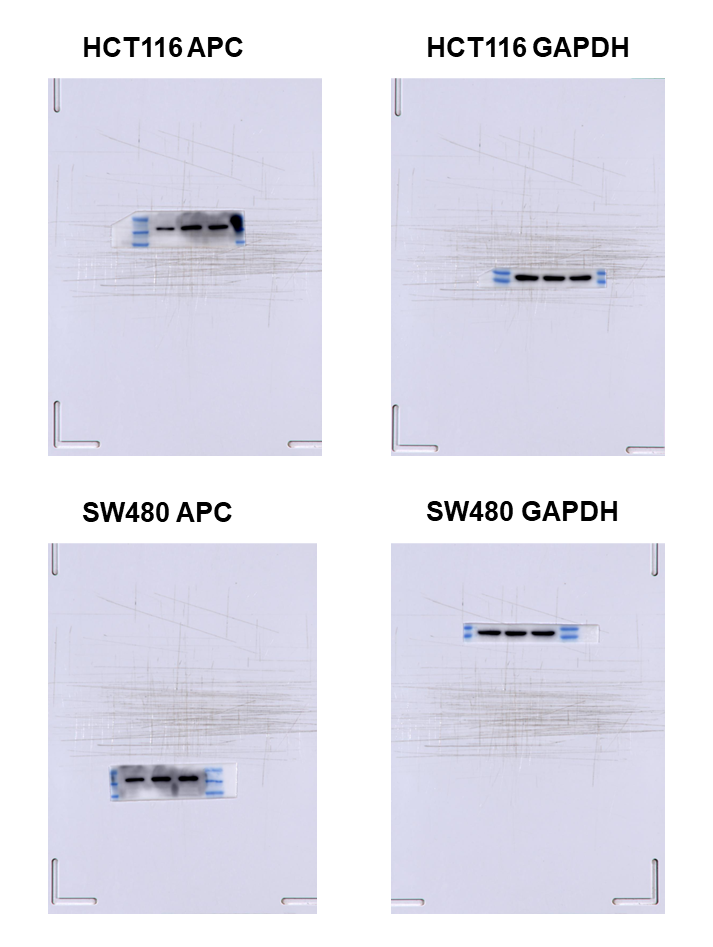


**Supplemental Figure 2: The APC expression of si-SLC7A7 was analyzed by WB in HCT116 and SW480 cells**


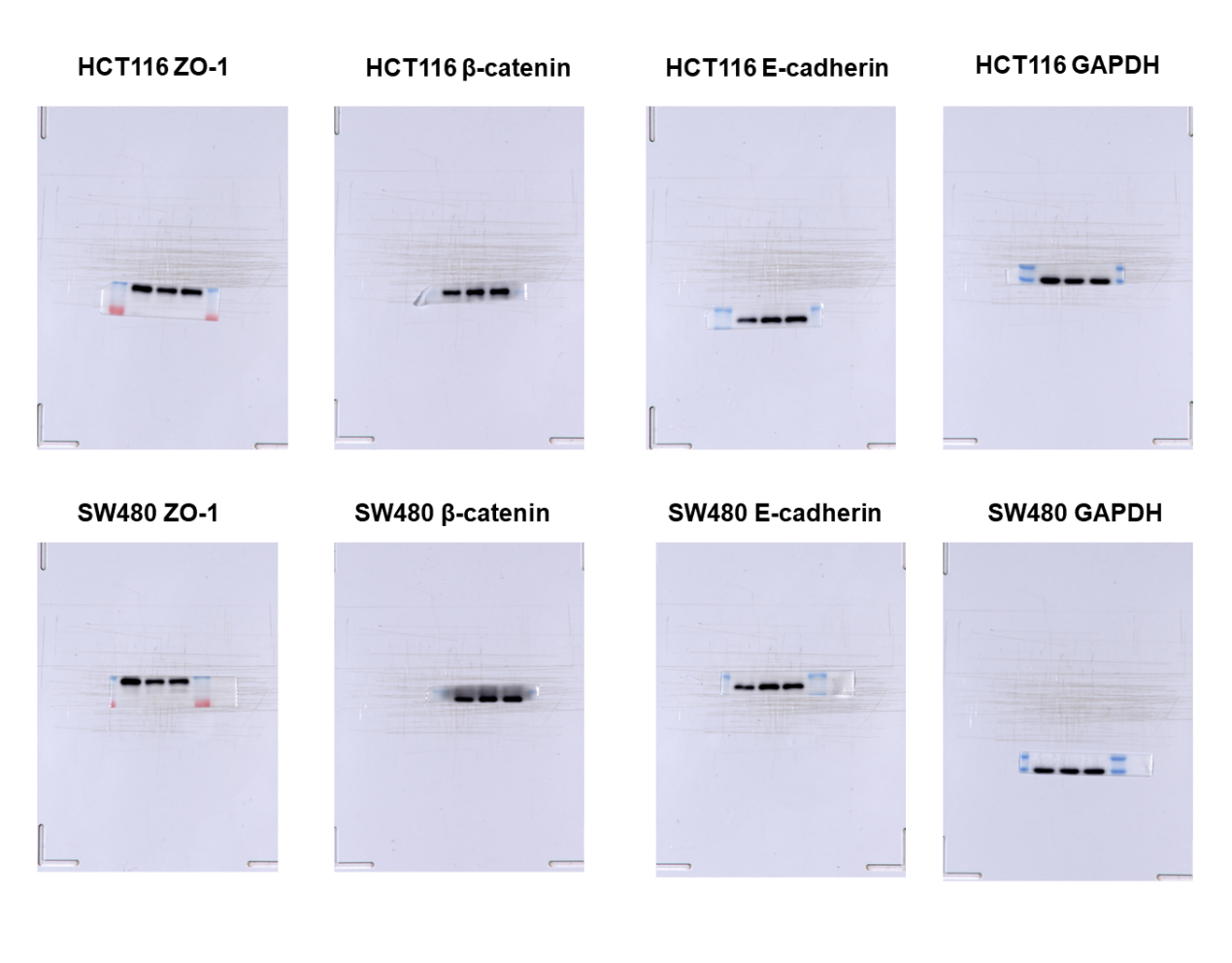


**Supplemental Figure 3: EMT marker molecular WB raw images**


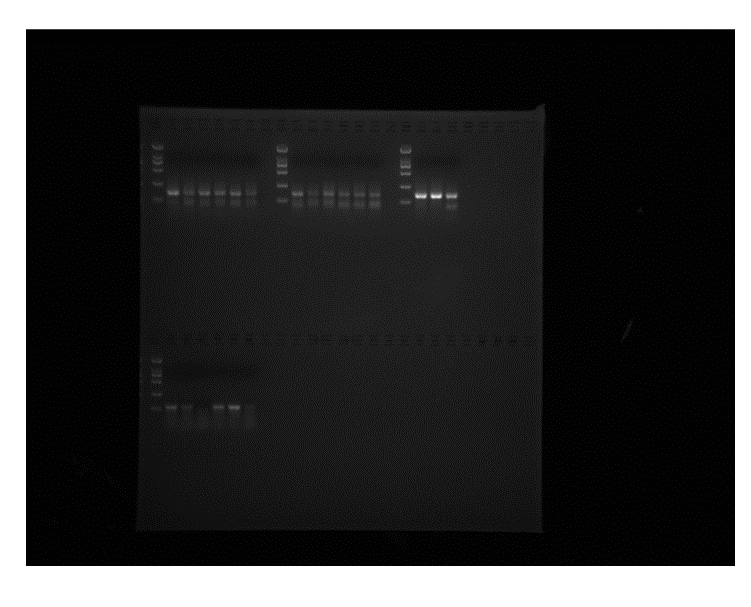


**Supplemental Figure 4: M7G Nucleic Acid Gel Imaging**

The upper half of the image shows the results of other experiments, and the lower half shows the results of the SLC7A7 m7G rip experiment, SW480/HCT116 two colon cancer cell line results.


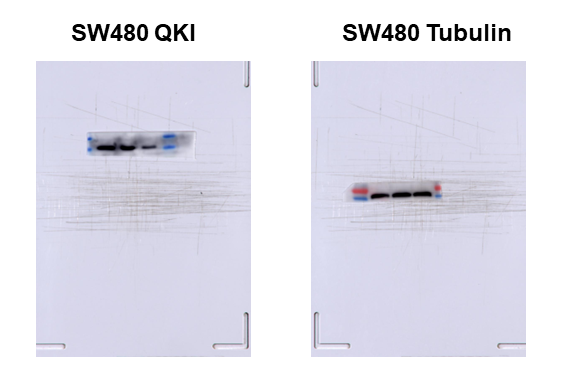


**Supplemental Figure 5: The efficiency of QKI siRNA was detected using WB in SW480 cells**
